# Low‐Titre ANA Late‐Onset Systemic Lupus Erythematosus Presenting With Pleural Effusion and Markedly Elevated Pleural Adenosine Deaminase Mimicking Tuberculous Pleuritis

**DOI:** 10.1002/rcr2.70785

**Published:** 2026-11-02

**Authors:** Shigesato Inoue, Miyoko Tatsuta, Tomofumi Tatsutani, Sho Ueda, Kentaro Wakamatsu, Masayuki Kawasaki

**Affiliations:** ^1^ Department of Respiratory Medicine National Hospital Organization Omuta National Hospital Omuta Fukuoka Japan; ^2^ Department of Rheumatology National Hospital Organization Omuta National Hospital Omuta Fukuoka Japan; ^3^ Department of Medicine and Biosystemic Science, Graduate School of Medical Sciences Kyushu University Fukuoka Japan

**Keywords:** adenosine deaminase, autoimmune disease, pleural effusion, systemic lupus erythematosus, tuberculous pleuritis

## Abstract

Pleural effusion with markedly elevated adenosine deaminase (ADA) levels is commonly associated with tuberculous pleuritis but may occur in other conditions. We report a case of low‐titre ANA late‐onset systemic lupus erythematosus (SLE) presenting with pleural effusion and markedly elevated ADA levels mimicking tuberculosis. A 78‐year‐old man with pneumoconiosis presented with fever, cough, and progressive pleural effusion. Pleural fluid analysis revealed lymphocyte‐predominant exudative effusion with markedly elevated ADA levels. Tuberculous pleuritis was initially suspected, but microbiological tests were negative. The patient developed thrombocytopenia, renal dysfunction, and altered consciousness. Immunological findings showed hypocomplementemia and elevated anti–double‐stranded DNA antibodies despite low‐titre ANA. Corticosteroid therapy led to rapid improvement, confirming the diagnosis of late‐onset SLE. Clinicians should consider autoimmune diseases when tuberculosis is not microbiologically confirmed.

## Graphical abstract

Pleural effusion with markedly elevated adenosine deaminase levels usually suggests tuberculous pleuritis. We report a case of low‐titre ANA late‐onset systemic lupus erythematosus presenting with lymphocyte‐predominant pleural effusion and markedly elevated ADA levels mimicking tuberculosis. Clinicians should consider autoimmune diseases such as SLE when microbiological evidence of tuberculosis is lacking.

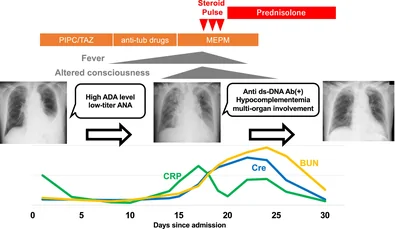

## 1 Introduction

Pleural effusion is a common clinical problem, and tuberculous pleuritis is an important cause. Measurement of adenosine deaminase (ADA) levels in pleural fluid is widely used as a diagnostic tool because of its high sensitivity for tuberculous pleuritis. Several studies have demonstrated that elevated ADA levels are strongly associated with tuberculous pleural effusion, making it a useful diagnostic marker in clinical practice [1]. However, elevated ADA levels are not specific to tuberculosis and may also be observed in several non‐tuberculous conditions, including lymphoma, rheumatoid pleuritis, empyema, and autoimmune diseases [2].

Systemic lupus erythematosus (SLE) is a multisystem autoimmune disease that can involve serosal membranes, and pleural effusion is a recognized manifestation of the disease. However, the majority of pleural effusions with markedly elevated ADA levels are caused by tuberculosis, whereas autoimmune diseases such as SLE represent a relatively uncommon cause. In addition, late‐onset SLE in elderly patients often presents with atypical clinical features and may mimic infectious diseases, which can delay diagnosis [3]. Although most patients with SLE present positive antinuclear antibody (ANA) tests, rare cases of SLE with low‐titre ANA have been reported [4]. Here, we report a case of low‐titre ANA late‐onset SLE presenting with lymphocyte‐predominant pleural effusion and markedly elevated ADA levels in a patient with pneumoconiosis.

## 2 Case Report

A 78‐year‐old man with pneumoconiosis under regular follow‐up developed progressive pleural effusion, fever, and cough in late November and was admitted to our hospital for further evaluation. Thoracentesis was performed (Table 1), and cytological examination of the pleural fluid was negative for malignancy. Empirical antibiotic therapy with piperacillin/tazobactam was initiated while further investigations were conducted.

**TABLE 1:** Pleural fluid analysis.

Parameter	Value	Unit
Specific gravity	1.030	
pH	7.1	
Total cell count	1564	cells/μL
Differential cell count		
Neutrophils	1	%
Lymphocytes	92	%
Macrophages	7	%
Total protein	4.5	g/dL
Glucose	88	mg/dL
Albumin	1.9	g/dL
Rivalta test	Positive	
ADA	110.9	U/L
Hyaluronic acid	30,200	ng/mL

*Note:* Pleural fluid examination demonstrated lymphocyte‐predominant exudative effusion with markedly elevated adenosine deaminase (ADA) levels.

Repeated sputum examinations showed no significant bacterial growth, and acid‐fast bacilli smears were negative. Chest computed tomography demonstrated well‐defined nodular opacities predominantly in both upper lobes, patchy infiltrates predominantly in the lower lobes, and bilateral pleural effusions, without findings suggestive of active pulmonary tuberculosis (Figure 1a,b). Interferon‐γ release assays, including QuantiFERON‐TB and T‐SPOT.TB, were negative. However, pleural fluid analysis revealed markedly elevated adenosine deaminase (ADA) levels (110.9 U/L), and a lymphocyte‐predominant exudative effusion (92% lymphocytes). Both pleural fluid and sputum were negative for acid‐fast bacilli on smear and polymerase chain reaction testing. Although the antinuclear antibody (ANA) titre was within the institutional reference range (titre 1:40), which is considered a low‐titre, diagnostic anti‐tuberculosis therapy with rifampicin, isoniazid, and ethambutol was initiated on Day 8 because tuberculous pleuritis could not be excluded, particularly given the markedly elevated ADA level and the upcoming long holiday period.

**FIGURE 1:**
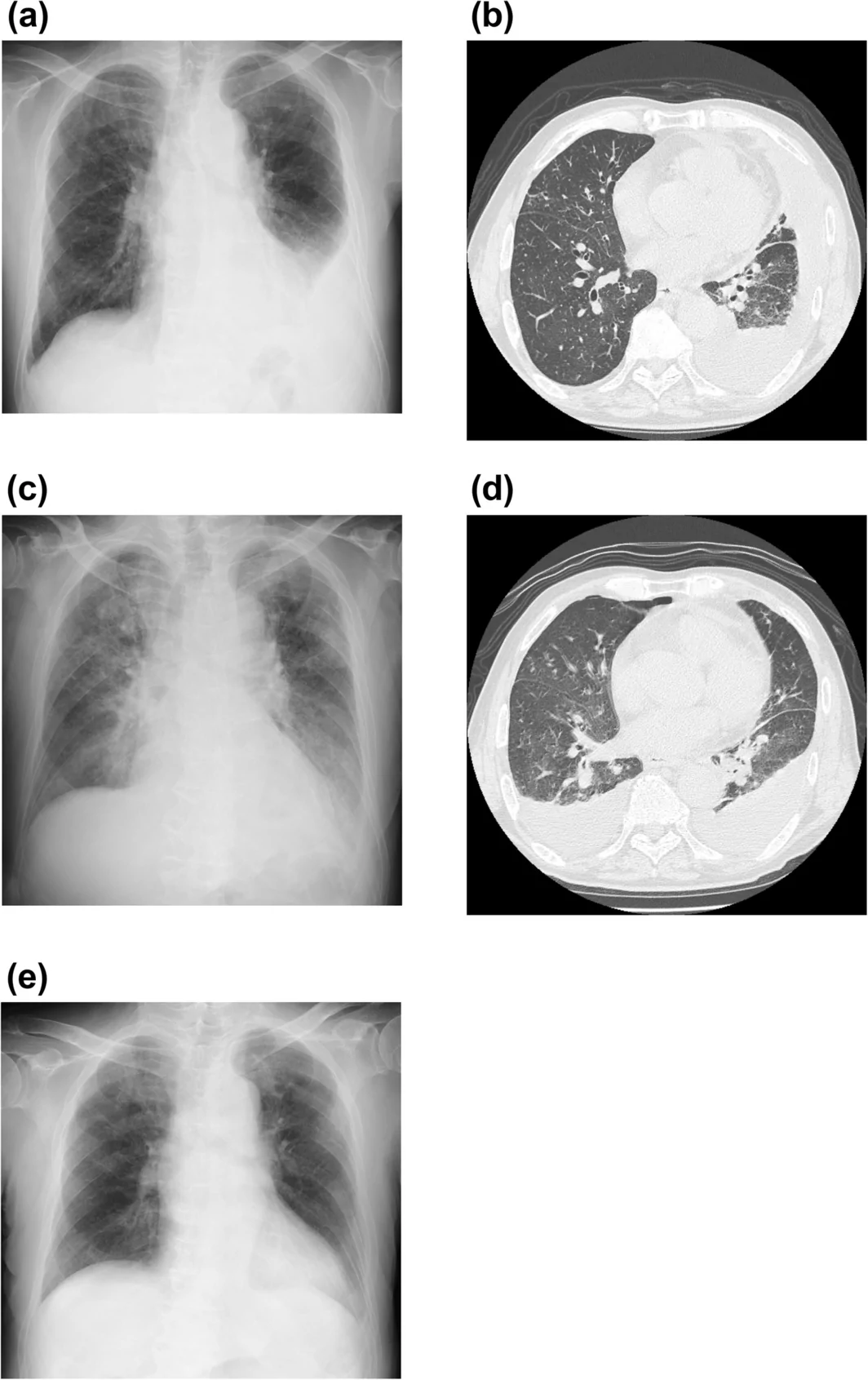
Serial chest imaging during the clinical course. (a, b) Chest radiograph and chest computed tomography (CT) obtained on admission showing left‐sided pleural effusion. (c, d) Chest radiograph and CT obtained during clinical deterioration (Day 17), demonstrating progression of pleural effusion. (e) Chest radiograph obtained after corticosteroid therapy (Day 57), showing marked resolution of pleural effusion.

On Day 13, the patient developed altered consciousness. Blood tests on Day 15 showed thrombocytopenia and evidence of hepatic and renal dysfunction (Table 2). Persistent fever was noted. Laboratory evaluation showed ferritin at 524 ng/mL and fibrinogen at 152 mg/dL. Computed tomography demonstrated worsening pleural effusion (Figure 1c,d). However, splenomegaly was absent. Because a skin rash appeared during treatment, drug hypersensitivity syndrome was suspected, and anti‐tuberculosis therapy was discontinued. Given thrombocytopenia and markedly elevated fibrin degradation products (201 μg/mL), disseminated intravascular coagulation associated with sepsis was suspected, and treatment with meropenem and recombinant thrombomodulin was initiated.

**TABLE 2:** Laboratory findings during hospitalization.

Day	1	8	13	15	17	20	26	30	
WBC	5200	2700	3500	3000	3600	5200	4100	4800	cells/μL
Neutrophils	3567	1871	3035	2427	3064	4612	3551	4032	cells/μL
Hb	14.1	12.7	14.0	14.0	13.6	13.0	11.7	11.4	g/dL
Plt	13.1	11.4	5.6	4.3	4.9	5.2	2.7	3.2	×10^4 cells/μL
Total bilirubin	1.2	1.0	1.0	0.9	0.4	0.5	0.5	0.4	mg/dL
AST	35	46	55	312	347	226	90	96	U/L
ALT	16	23	54	66	51	49	26	43	U/L
γ‐GTP	31	42	764	114	84	90	54	50	U/L
LDH	277	293	328	1347	2037	1598	1011	839	U/L
CRP	5.00	0.49	2.34	3.95	6.56	1.49	2.24	0.66	mg/dL

*Note:* Serial laboratory findings showing thrombocytopenia, elevated lactate dehydrogenase levels, and transient hepatic and renal dysfunction during the clinical course.

Despite these interventions, the patient's respiratory condition deteriorated by Day 17, requiring high‐flow nasal cannula oxygen therapy (flow 30 L/min, FiO_2_ 60%). Lactate dehydrogenase levels increased further, and renal dysfunction worsened. Although ANA remained low‐titre, immunological testing revealed hypocomplementemia (C3 35 mg/dL, C4 3 mg/dL), positive anti‐RNP antibodies (32.8 U/mL), positive anti‐SS‐A antibodies (138 U/mL), and markedly elevated anti–double‐stranded DNA antibodies (353 IU/mL). Based on these findings, late‐onset systemic lupus erythematosus was clinically diagnosed despite not fulfilling the ANA entry criterion of the 2019 ACR/EULAR classification criteria.

Given the rapidly progressive clinical deterioration, transfer to a tertiary care center for advanced supportive therapy, including continuous haemodiafiltration, was considered. However, the patient's family declined transfer. High‐dose corticosteroid pulse therapy was initiated on the same day (Figure 2). The pleural effusion improved rapidly following steroid treatment, and renal dysfunction subsequently improved. His respiratory status also improved, with oxygen saturation reaching 97% on room air.

**FIGURE 2:**
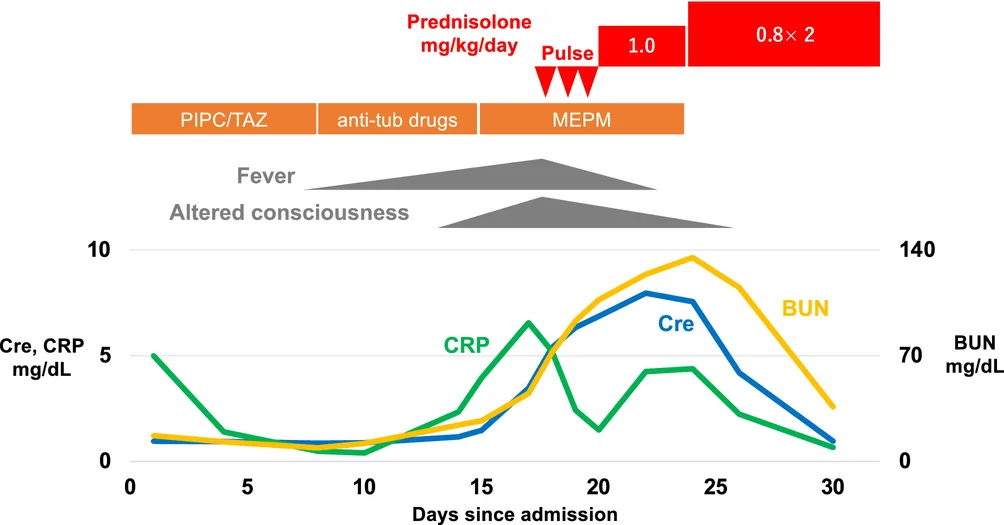
Clinical course of the patient. The clinical course during hospitalization is shown. Anti‐tuberculosis therapy was initiated because tuberculous pleuritis was suspected based on the markedly elevated pleural ADA level. The patient subsequently developed altered consciousness, thrombocytopenia, and renal dysfunction. After initiation of corticosteroid pulse therapy, pleural effusion and organ dysfunction rapidly improved.

Considering the need for further immunosuppressive therapy under specialist care, the patient was transferred to a tertiary care hospital on Day 31. Subsequent cultures later yielded *Mycobacterium intracellulare* from a sputum specimen, whereas pleural fluid cultures remained negative. The isolate was considered colonization rather than the primary cause of pleuritis. At the tertiary care center, ANA remained low‐titre (1:40), whereas anti–double‐stranded DNA antibodies remained positive with persistent hypocomplementemia, supporting the diagnosis of SLE. The patient was later transferred to a rehabilitation facility on Day 57 (Figure 1e).

## 3 Discussion

The present case highlights several important clinical lessons regarding the differential diagnosis of pleural effusion with elevated ADA levels.

First, markedly elevated pleural ADA levels do not necessarily indicate tuberculous pleuritis. ADA is an enzyme involved in purine metabolism and is particularly abundant in activated lymphocytes. Because tuberculous pleuritis is characterized by intense cellular immune responses with lymphocyte predominance, pleural ADA levels are frequently elevated and have been widely used as a diagnostic marker for tuberculosis [1]. Previous studies have shown that higher ADA levels are associated with a greater likelihood of tuberculosis. In the present case, the ADA level of 110.9 U/L was well above this range, which strongly supported the initial clinical suspicion of tuberculous pleuritis. However, ADA elevation is not specific to tuberculosis. Increased ADA levels have also been reported in other conditions associated with lymphocyte‐rich pleural effusion, including lymphoma, rheumatoid pleuritis, empyema, and autoimmune diseases such as SLE. Therefore, although pleural ADA levels exceeding 40 U/L are commonly used as a diagnostic cutoff for tuberculous pleuritis, clinicians should interpret these findings cautiously in the context of the overall clinical picture. In the present case, tuberculous pleuritis was initially suspected because of the markedly elevated ADA level; however, repeated microbiological tests, including acid‐fast smear, PCR, and culture of pleural fluid were negative, and the pleural effusion improved rapidly after corticosteroid therapy. These findings supported a non‐tuberculous aetiology.

Second, this case illustrates the diagnostic challenges associated with late‐onset SLE. SLE typically affects young women; however, approximately 10%–20% of patients develop the disease after the age of 50 years and are classified as having late‐onset SLE [3]. Compared with younger patients, late‐onset SLE often presents with atypical manifestations and less prominent mucocutaneous findings. Serositis, including pleural effusion, may be one of the initial manifestations and may resemble infectious diseases. In the present case, the absence of typical mucocutaneous manifestations and the initial presentation with serositis and systemic inflammation were consistent with the reported characteristics of late‐onset SLE.

Another notable feature of this case was the absence of significant ANA positivity. The 2019 ACR/EULAR classification criteria require ANA positivity as an entry criterion for SLE classification; however, low‐titre ANA SLE has been reported in rare cases. In the present case, despite low‐titre ANA results, the patient showed markedly elevated anti–double‐stranded DNA antibodies and hypocomplementemia, together with multi‐organ involvement, and a dramatic response to corticosteroid therapy, all of which supported the diagnosis of SLE.

Importantly, the patient fulfilled the 2012 Systemic Lupus International Collaborating Clinics (SLICC) classification criteria, with leukopenia, thrombocytopenia, serositis, hypocomplementemia, and anti–double‐stranded DNA antibody positivity. These findings further supported the classification of SLE despite the absence of significant ANA positivity. Finally, the presence of pneumoconiosis in this patient may also be relevant. Exposure to silica dust has been associated with an increased risk of autoimmune diseases, including SLE, rheumatoid arthritis, and systemic sclerosis [5]. Epidemiological studies have also suggested that occupational exposure to crystalline silica increases the risk of developing SLE. Silica particles may activate the innate immune system and promote autoantibody production, potentially contributing to autoimmune disease development.

Clinicians should be aware that markedly elevated pleural ADA levels are not pathognomonic for tuberculosis and may lead to diagnostic delay if other causes, including autoimmune diseases such as SLE, are overlooked.

## Author Contributions

Shigesato Inoue prepared the manuscript, which was reviewed by all co‐authors. All authors have approved the final version of the manuscript for submission.

## Funding

The authors have nothing to report.

## Ethics Statement

The authors declare that appropriate written informed consent was obtained for the publication of this manuscript and accompanying images.

## Consent

The authors declare that written informed consent was obtained for the publication of this manuscript and accompanying images and attest that the form used to obtain consent from the patient complies with the Journal requirements as outlined in the author guidelines.

## Conflicts of Interest

The authors declare no conflicts of interest.

## Data Availability

The data that support the findings of this study are available from the corresponding author upon reasonable request.
